# Refining Pure-Tone Average Calculations for Reporting Hearing Outcomes: Advocating a Shift to the Logarithmic Mean

**DOI:** 10.1055/s-0045-1801853

**Published:** 2025-11-11

**Authors:** Ali Faramarzi

**Affiliations:** 1 Department of Otolaryngology, Otolaryngology Research Center, Shiraz University of Medical Sciences, Shiraz, Iran; 2 Student Research Committee, Shiraz University of Medical Sciences, Shiraz, Iran

Dear Editor,

Uniformity in reporting hearing outcomes is paramount for accurate evaluation and comparison of hearing-related research. Standardized guidelines are essential as, without them, comparing data between studies becomes problematic. One widely accepted method is the 4-frequency pure-tone average (PTA), using frequencies of 0.5, 1, 2, and 3 kHz. This approach was first recommended in the 1979 American Medical Association (AMA) guidelines for calculating hearing handicap. 1 Subsequently, the AMA's chosen frequencies were incorporated into the 1995 2 3 and 2012 4 guidelines issued by the Hearing and Equilibrium Committee of the American Academy of Otolaryngology—Head and Neck Surgery (AAO-HNS). These guidelines for reporting hearing results are still recommended and used today. 2 3 4

Traditionally, the calculation of PTA has followed the guidelines set forth by renowned institutions such as the AMA 1 and the AAO-HNS. 2 3 4 These bodies initially proposed the simple arithmetic mean (SAM) as the method for calculating PTA. 1 However, considering the decibel (dB) scale's inherent logarithmic structure, 5 this may not accurately represent PTA values. This understanding necessitates a critical reevaluation of current practices, suggesting that a logarithmic mean might yield more accurate results than the SAM. For instance, on a logarithmic scale, the average of 10 and 30 dB is not 20 dB but approximately 27.03 dB. This proposed analytical shift, aligning with the dB scale's inherent logarithmic nature, could potentially facilitate a more precise determination of PTA. The steps to achieve this are as follows:

1. **Conversion to linear scale:** This initial step involves converting each dB value to a linear scale using the formula: **P _linear_** = **10 ^(P^_dB_^/10)^** . This conversion is vital, as dB is a logarithmic measure of sound intensity.
2. **Arithmetic mean calculation:** After conversion, the arithmetic mean of the linear scale values is calculated by adding all the converted values and then dividing by the total count.
3. **Conversion back to dB scale:** The final step is to convert the mean value back to the dB scale, which can be done using the formula: P _dB_ = 10*Log _10_ (P _linear_ ) .

The reevaluation of hearing outcome reporting guidelines 1 2 3 4 shows that the recommended PTA calculation formula is the SAM, which might be inappropriate for a logarithmic measure such as the dB scale. This underlines the importance of accurate and appropriate reporting of PTAs, since such data plays a pivotal role in diagnosis and treatment decisions. For greater accuracy, a shift from the SAM to the logarithmic mean should be considered.

While it is acknowledged that implementing new standards often requires a transition period, it is essential to recognize that the proposed formula utilizes routinely collected data, merely suggesting a different approach to calculating averages. Consequently, the transition period is expected to be relatively brief. Hence, a thoughtful revision of the guidelines on reporting hearing outcomes is needed. This modification, which is essential for the sake of accuracy, can be implemented effectively. Thus, we can bring about improvements in the quality and consistency of data reporting in hearing healthcare, thereby enabling a more precise and meaningful comparison between studies.
